# Voluntary wheel running in mice increases the rate of neurogenesis without affecting anxiety-related behaviour in single tests

**DOI:** 10.1186/1471-2202-13-61

**Published:** 2012-06-08

**Authors:** Lillian Garrett, D Chichung Lie, Martin Hrabé de Angelis, Wolfgang Wurst, Sabine M Hölter

**Affiliations:** 1 Institute of Developmental Genetics, Helmholtz Zentrum München, German Research Center for Environmental Health, Neuherberg/Munich, Germany; 2 Institute of Experimental Genetics, Helmholtz Zentrum München, German Research Center for Environmental Health, Neuherberg/Munich, Germany; 3 German Mouse Clinic, Helmholtz Zentrum München, German Research Center for Environmental Health, Neuherberg/Munich, Germany; 4 Technische Universität München, Lehrstuhl für Entwicklungsgenetik, Freising-Weihenstephan, Germany; 5 Technische Universität München, Lehrstuhl für Experimentelle Genetik, Freising-Weihenstephan, Germany; 6 Deutsches Zentrum für Neurodegenerative Erkrankungen e. V. (DZNE), Site Munich, Germany; 7 Max Planck Institute of Psychiatry, Munich, Germany

## Abstract

**Background:**

The role played by adult neurogenesis in anxiety is not clear. A recent study revealed a surprising positive correlation between increased anxiety and elevated neurogenesis following chronic voluntary wheel running and multiple behavioural testing in mice, suggesting that adult hippocampal neurogenesis is involved in the genesis of anxiety. To exclude the possible confounding effect of multiple testing that may have occurred in the aforementioned study, we assessed (1) the effects of mouse voluntary wheel running (14 vs. 28 days) on anxiety in just one behavioural test; the open field, and (2), using different markers, proliferation, differentiation, survival and maturation of newly born neurons in the dentate gyrus immediately afterwards. Effects of wheel running on anxiety-related behaviour were confirmed in a separate batch of animals tested in another test of anxiety, the light/dark box test.

**Results:**

Running altered measures of locomotion and exploration, but not anxiety-related behaviour in either test. 14 days running significantly increased proliferation, and differentiation and survival were increased after both running durations. 28 day running mice also exhibited an increased rate of maturation. Furthermore, there was a significant positive correlation between the amount of *proliferation*, but not maturation, and anxiety measures in the open field of the 28 day running mice.

**Conclusions:**

Overall, this evidence suggests that without repeated testing, newly born *mature neurons* may not be involved in the genesis of anxiety *per se*.

## Background

Adult mammalian hippocampal neurogenesis is the birth of new neurons from neural precursor cells in the subgranular zone of the dentate gyrus [1-3]. This process can be subdivided into a series of distinct phases that begins with proliferation of Type II neural precursor cells, followed by neuronal lineage specification, migration, maturation and synaptic integration into existing hippocampal neurocircuitry [4]. The precise functional role of these newly generated granule cells is not known [5]. While much of the available research has focused on their role in learning and memory [6-8], there is also evidence linking neurogenesis to emotion-related behaviours such as anxiety [6,9-11]. The widely held view is that decreased neurogenesis is associated with increased anxiety [12-14], with some evidence to the contrary [6,8,15].

A frequently used means to experimentally increase neurogenesis in mice is voluntary wheel running/exercise [3,16]. Few studies have directly tested the relationship between exercise-induced neurogenesis and anxiety-related behaviour. A recent investigation was one of the first to explore this association [17]. The outcome revealed that three to four weeks running caused a surprising increase in anxiety-related behaviour in mice that positively correlated with elevated measures of neurogenesis. The conclusion that increased neurogenesis plays a role in the genesis of anxiety is in opposition to the widely held view. Nevertheless, this study involved an extensive behavioural test battery, the stressful nature of which, as well as the prolonged period of time before animals were sacrificed (in total 45 days), could have affected neurogenesis. Furthermore, the experience of multiple test exposures and handling involves learning and might have affected behavioural measures [18]. In the current study, we addressed these confounding issues and directly asked the question whether exercise-induced neurogenesis increases anxiety responding without any prior experience. To this end we looked at the effects of 14 and 28 days voluntary wheel running in mice on just one set of behaviours; those in the open field test. The animals were sacrificed immediately after to avoid the confounding effect of repeated testing and to assess the different phases of neurogenesis (proliferation, survival, differentiation and maturation) at the time point of testing. To confirm the effects of wheel running on anxiety-related behaviour this experiment was repeated in a separate batch of animals, but instead of the open field the animals were tested in another test for anxiety, the light/dark box test.

## Methods

### Animals

Male C57BL/6 J mice (cohort 1: n = 39, cohort 2: n = 20) were obtained at 7 weeks of age from the animal breeding unit of the Helmholtz Zentrum München. Upon their arrival, animals were singly housed in individually ventilated cages in a temperature (22 - 24°C) and humidity (50 – 60%) controlled environment on a 12/12 h light/dark cycle (lights on at 7 am). Water and food were available ad libitum. All experiments were carried out in accordance with the European Communities Council Directive of 24 November 1986 (86/609/EEC) and approved by the responsible animal welfare authority (Regierung von Oberbayern).

### Voluntary wheel running

**Figure 1:**
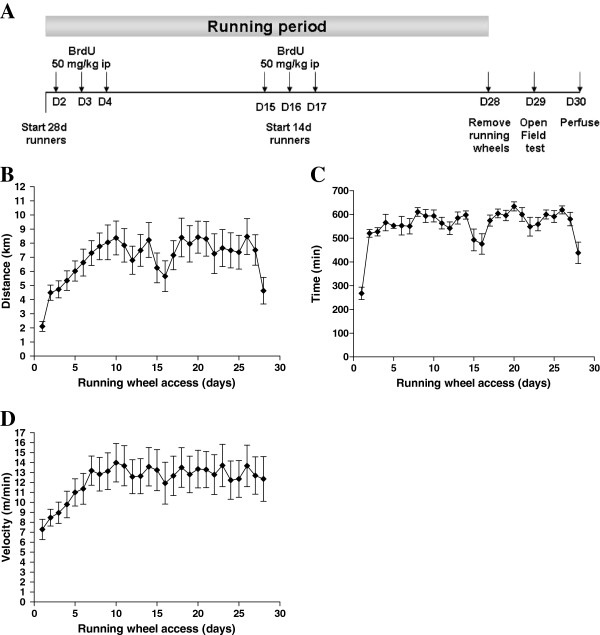
**(A) Experimental timeline (see methods for details; D = day) and (B) average distance run, (C) average amount of time spent running and (D) average velocity of running per 24 h over 4 weeks of running wheel access.** Data represent means ± S.E.M.

The experimental timeline is illustrated in Figure 1A. After a 1 week acclimatisation period, mice randomly assigned to the 14- and 28-day running groups (n = 16 and 14 respectively) were provided with an angled running wheel (diameter 15.50 cm) complete with a wire-less controlled activity counter (Wheel Manager software, Med Associates Inc., VT, USA). These time-points were chosen as changes in anxiety-related behaviour had previously been shown after this amount of exercise [19,20] and also newly born neurons have functionally matured by the 28 day time-point [21]. Running wheels were removed 24 h prior to behavioural testing. Sedentary control animals (n = 14–15 per group) were singly housed for the duration of the experiment without a running wheel. As an additional measure of anxiety-related behaviour, a separate batch of C57BL/6 J mice (n = 10 sedentary controls, n = 10 wheel runners) were subjected to the same experimental sequence but exposed to the light/dark box test on completion of 28 days running. To exclude the possible confounding effect of wheel removal the day before behavioural testing, which may be stressful for the animal because it is a change in their environment, an additional blocked wheel control group (n = 10) was included.

### BrdU injection

5’-bromo-2’-deoxyuridine (BrdU) (Sigma, Germany) was dissolved in a 0.9% (w/v) NaCl solution. After one day running, experimental animals and controls were injected with 50 mg/kg BrdU intra-peritoneally once a day for 3 days.

### Open field analysis

On completion of the respective running periods, all animals were tested the next day during the light phase using the open field test, which consisted of a transparent and infra-red light permeable acrylic test arena with a smooth floor (internal measurements: 45.5 x 45.5. x 39.5 cm). Illumination levels were set at approx. 150 lux in the corners and 200 lux in the middle of the test arena. Data were recorded and analysed using the ActiMot system (TSE, Bad Homburg, Germany).

### Light/dark box analysis

The test box was made of PVC and divided into two compartments, connected by a small tunnel (4 x 6 x 9 cm high). The lit compartment (29 x 19 x 24 cm high) was made of transparent PVC and was illuminated by cold light with an intensity of 650 lux in the middle; the dark compartment (14 x 19 x 24 cm high) was made of black PVC and not directly illuminated (approx. 20 lux in the centre). The mouse was placed in the centre of the black compartment and allowed to freely explore the apparatus for 5 min. Data were recorded and analysed using the ActiMot system (TSE, Bad Homburg, Germany).

### Tissue preparation

The day after open field and light/dark box analysis, animals were deeply anaesthetised using CO_2_ and perfused transcardially with 4% paraformaldehyde (PFA) in 0.1 M phosphate buffer. Brains were dissected from the skulls, post-fixed overnight in 4% PFA at 4°C and then transferred to a 30% (w/v) sucrose solution until saturated. Brains were then cut on a dry ice-cooled block with a sliding microtome (Leica, Bensheim) into 40 μm-thick coronal free-floating sections and stored at −20°C in a cryoprotectant solution containing 25% ethylene glycol and 25% glycerine in phosphate buffer. A one-in-six series of sections was taken for analysis from the brains of a subset of animals from each group.

### Immunostaining

For epifluorescent detection of Ki67, BrdU and Calbindin, a protocol similar to that implemented in previous studies was employed [22,23]. For Ki67 staining, a primary polyclonal anti-Ki67 antibody (rabbit anti-Ki67-antibody; 1:250; NCL-Ki67p, Novacastra, Newcastle upon Tyne, UK) was used with a secondary goat anti-rabbit IgG (Cy3 labelled; 1:250, Jackson Immunoresearch Laboratories Inc. West Grove, PA, USA). Sections were counterstained with the fluorescent DNA marker 4’,6-diamidino-2’-phenylindole-dihydrochloride (DAPI in TBS; 1:10,000; Sigma, Germany). For BrdU and calbindin staining, free-floating sections were stained with anti-BrdU (1:400, rat anti-BrdU, AbD Serotec, Raleigh, NC) and anti-Calbindin (1:2000; rabbit monoclonal anti-Calbindin D-28 k, SWANT, Bellinzona, Switzerland) antibodies. Secondary antibodies used were donkey anti-rat IgG (Cy3 labelled, 1:400; Jackson Immunoresearch Inc.) and donkey anti-rabbit IgG (Cy2 labelled; 1:300; Jackson Immunoresearch Inc.). For the immunoperoxidase detection of doublecortin, a procotol similar to that employed by Rao and Shetty [24] was used. A primary goat polyclonal anti-DCX antibody (Doublecortin; 1:200, sc-8066, Santa Cruz Biotechnology, Santa Cruz, CA) was used with a biotinylated rabbit anti-goat IgG (1:300; Jackson Immunoresearch Laboratories Inc.) and 3,3’-diaminobenzidine (DAB) as the chromogen.

### Quantification of BrdU- and Ki67-positive cells

To quantify the number of BrdU- and Ki67-positive cells across the rostro-caudal extent of the dentate gyrus, a modified stereological approach was employed similar to that described [25].

### Immunofluorescence analysis

Fluorescent signals were imaged in z-stacks at 1 μm intervals using Zeiss Axiovert confocal laser scanning microscope 510 with a 40x objective. Fifty BrdU-positive cells within the subgranular zone and the granule cell layer were analysed for co-expression of BrdU and the neuronal maturation marker calbindin and percentages were determined. This was to determine the proportion of BrdU + cells that differentiated into neurons.

### Quantification of DCX-positive neurons

Estimation of the total number of DCX-positive neurons was determined using unbiased stereology with the optical fractionator method and the semiautomatic StereoInvestigator system (MicroBrightField Inc., Williston, VT, USA). For this purpose, the granule cell layer of the hippocampal dentate gyrus was traced in every 6^th^ section and the reference volume was determined. Immunopositive cells were quantified by systematic random sampling using a scan grid size of 200x200 μm and a counting frame of 100x100 μm. Cells that intersected the uppermost focal plane or the lateral exclusion borders of the counting frame were not quantified.

### Statistical analysis

Numerical analyses were performed using SPSS software (SPSS Inc, Chicago, USA) and GraphPad Prism software. One- and two-way analysis of variance (ANOVA) were used, followed by one-tailed post-hoc tests (Tukey’s and Bonferroni’s). The Pearson’s correlation coefficient was used to establish correlations between running distances, behaviour and cell counts. Differences were deemed statistically significant at p < 0.05.

## Results

### Running behaviour

Mice began running an average of 2.10 km/night on day 1 and steadily increased activity each day towards an average peak of 8.37 km/night on day 10, generally reaching a plateau from this point (Figure 1B). Over the 28 day running period, the cumulative distances run were on average between 65.50 and 342.47 km. Mice were spending up to 619.29 minutes running per night with speeds of up to 13.50 m/min (Figure 1C and 1D).

### Open field behaviour

**Figure 2:**
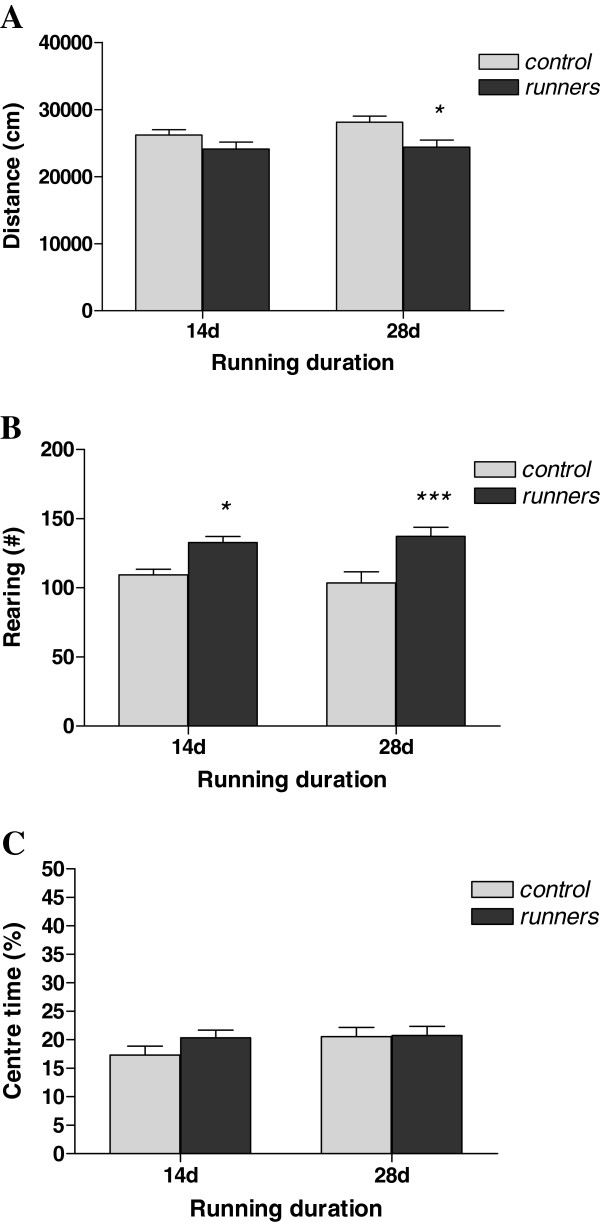
**Behaviour of running (14-day and 28-day) and control mice during a 20-min open field test.** In (**A**), (**B**) and (**C**), locomotor activity (distance moved), exploration (rearing) and anxiety-related behaviour (% centre time) are represented respectively. *p < 0.05, ***p < 0.001 vs. corresponding control. *N* = 14–16 per group. Data represent means ± S.E.M.

The open field is a test of locomotor activity, exploration and anxiety-related behaviour. A two-way ANOVA revealed a significant main effect of treatment (F_(1,55)_ = 9.01, p < 0.01) without a main effect of time (F_(1,55)_ = 1.32, n.s.) or a significant treatment x time interaction effect (F_(1,55)_ = 0.77, n.s.) on locomotor activity (indexed by total distance travelled; Figure 2A). Post-hoc analysis revealed that 28-day running wheel mice displayed a statistically significant (p < 0.05) difference in locomotor activity across the 20-minute open field test compared to controls.

Concerning exploratory activity (rearing; Figure 2B) in the open field, a two-way ANOVA again revealed a significant main effect of treatment (F_(1,55)_ = 23.67, p < 0.001) in the absence of either a main effect of time (F_(1,55)_ = 0.01, n.s.) or an interaction (F_(1,55)_ = 0.79, n.s.). Both 14- and 28-day running mice exhibited significantly increased rearing activity as assessed using post-hoc Bonferroni’s test (14-day runners: p < 0.05; 28-day runners: p < 0.001). This difference was significant across all 5-minute subdivisions of the 20-minute test (data not shown).

No significant main effects of treatment (F_(1,55)_ = 1.11, n.s.), time (F_(1,55)_ = 1.42, n.s.) or an interaction (F_(1,55)_ = 0.85, n.s.) were found on total % time spent in the centre (index of anxiety-related behaviour; Figure 2C) of the open field using a two-way ANOVA.

### Effect of wheel running on behaviour in the light/dark box

**Figure 3:**
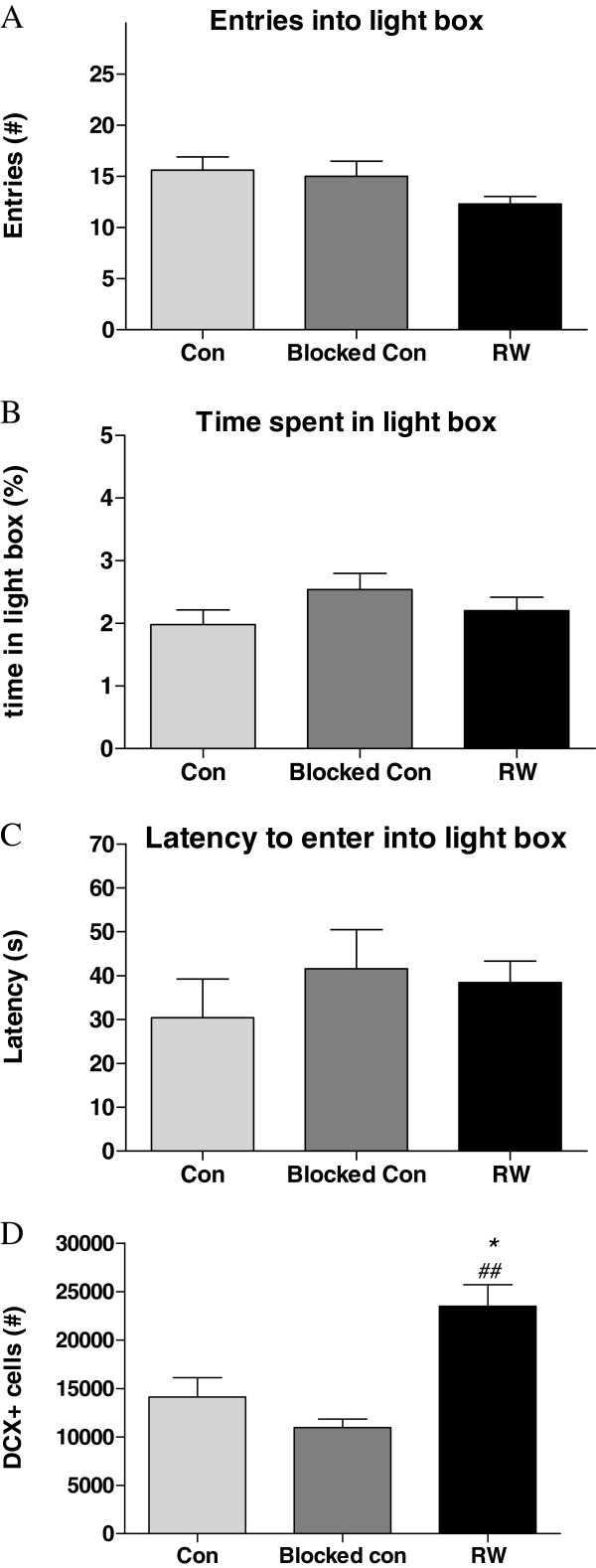
**(A) 14 days running caused significantly increased proliferating cell number (indexed by Ki67 immunoreactivity) in the dentate gyrus of male C57Bl/6 J mice, no longer present after 28 days.** (**B**) Marker for proliferation: fluorescent immunohistochemistry against Ki67 (red). (**C**) Both 14-days and 28-days of running increased survival (indexed by BrdU staining) in the dentate gyrus in these animals. (**D**) Representative photomicrograph showing a marker for survival: BrdU-immunoreactive cells in the dentate gyrus (red). (**E**) and (**F**) Running increases the number of new born neurons that reach maturation: 28-days running significantly increased the % of BrdU-labelled cells (red) that co-localised with calbindin (mature neuronal marker: green). (**G**) and (**H**) Both 14-days and 28-days running increased differentiation (indexed by DCX staining) of newly born cells into immature neurons in the dentate gyrus. (B + D) Scale bar = 50 μm (F) Scale bar = 20 μm (H) Scale bar = 70 μm Data represent means ± S.E.M. *p < 0.05, **p < 0.01, ***p < 0.001 vs. corresponding control.

In the light/dark box, a test of anxiety that exploits a rodent’s aversion to a brightly lit space, there were no significant effects of exercise on any of the anxiety-related parameters measured (Figure 3). This includes the main indices such as latency to enter light box (F_(2,27)_ = 0.55, n.s.); light box entries (F_(2,27)_ = 2.08, n.s.) and percentage time spent in light box (F_(2,27)_ = 1.42, n.s.).

### Effect of wheel running on proliferation of granule cell precursors

**Figure 4:**
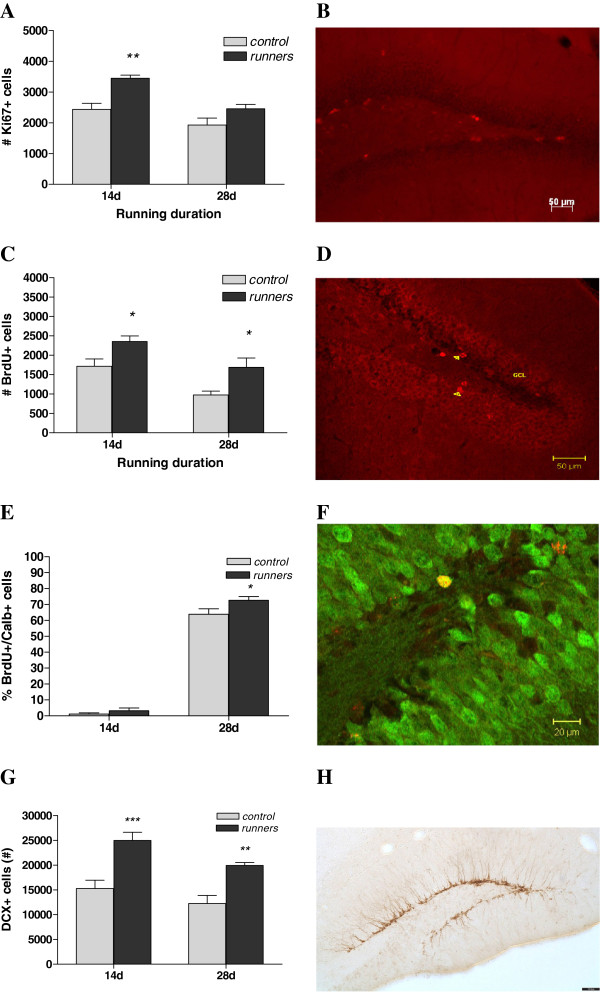
**Behaviour of running (28-day; RW) and control (Sedentary (con) and Blocked Wheel (blocked con)) mice during a 5-min light/dark box test.** In (**A**), (**B**) and (**C**), entries into light box, % time spent in light box and latency to enter light box are represented respectively. *N* = 10 per group. In (D) the total number of DCX positive cells in the dentate gyrus from the second cohort of animals in which the light/dark box was tested are shown. *p < 0.05 vs. con, ##p < 0.01 vs. blocked con. Data represent means ± S.E.M.

To determine the effects of the different running durations on the proliferation period of the neurogenic process, we quantified the numbers of Ki67+ cells in the subgranular zone/granule cell layer of the dentate gyrus, a protein present in proliferating cells [23] (Figure 4). A two-way ANOVA revealed significant main effects of treatment (F_(1,27)_ = 15.80, p < 0.001) and time (F_(1,27)_ = 15.00, p < 0.001) without an interaction (F_(1,27)_ = 1.57, n.s.). Post-hoc Bonferroni’s test found that the 14-day runners had a significantly increased number of Ki67-positive cells in the dentate gyrus compared to controls (p < 0.01). While 28-day runners still showed an increased number of proliferating cells compared to controls, this difference did not attain statistical significance.

### Effect of 2 and 4 weeks wheel running on the fate and rate of maturation of new neurons in the dentate gyrus

To assess the effects of the two wheel running durations on the fate of cells that proliferated after the initiation of running we labelled cells with BrdU for three days subsequent to commencement of running (Figure 4C and D). A two-way ANOVA found both a significant main effect of treatment (F_(1,38)_ = 15.80, p < 0.001) and time (F_(1,38)_ = 17.11, p < 0.001) without an interaction effect (F_(1,38)_ = 0.05, n.s.). An increased number of BrdU-positive cells in the dentate gyrus was seen after both 14 and 28 days of running in these mice as shown by post-hoc analysis (p < 0.05). This gives runners on average a 70.71% increase in BrdU-positive cells at the 28 day time-point. To assess the rate of maturation of new neurons in the dentate gyrus of running mice, the number of BrdU-positive cells that co-expressed the mature neuronal marker calbindin were quantified and compared between groups (Figure 4E and F). As expected (as it takes longer than 14 days for new neurons to become calbindin-positive) no significant difference was observed in the number of 14 day BrdU-positive neurons that were calbindin-positive. A *t*-test revealed a significant increase in the percentage of BrdU/calbindin positive cells in the dentate gyrus of 28-day running mice compared to controls (t_(17)_ = 2.18, p < 0.05; controls: 63.88 ± 3.40%; runners: 72.66 ± 2.30%).

### Effect of running on the number of DCX-positive neurons in the dentate gyrus

To assess the effect of wheel running on the differentiation of newly born cells, we estimated the total number of DCX-positive neurons in the hippocampal dentate gyrus. A two-way ANOVA revealed both a significant main effect of time (F_(1,35)_ = 7.32, p = 0.01) and exercise (F_(1,35)_ = 33.86, p < 0.0001). A Bonferroni’s test showed that both 14 and 28d runners had higher DCX-positive cells in the dentate gyrus compared to controls (Figure 4G and 4H). The number of DCX-positive cells was also quantified in the dentate gyrus of the separate cohort of animals exposed to the light/dark box. A one-way ANOVA revealed a significant difference between the groups (F(2,9) = 13.27, p = 0.002). A post-hoc Tukey’s test showed that the running wheel mice had higher DCX numbers compared to both the sedentary (p < 0.05) and blocked wheel (p < 0.01) control groups with the difference more pronounced in the latter (Figure 3D).

### Correlation analysis of running, open field behaviour and neurogenesis markers in exercising mice

Regression analysis between the measures of open field behaviour and neurogenesis in running mice revealed noteworthy correlations. In both the 14- and 28-day runners, there was a significant positive correlation between distance run on the running wheels and distance travelled in the open field (14d: r_p_ = 0.54, p < 0.05, 28d: r_p_ = 0.80, p = 0.01; Table 1). Concerning anxiety-related behaviour, in the 28-day runners there was a significant negative correlation between the numbers of Ki67+ cells in the dentate gyrus and the total % time spent in the centre of the open field (Table 1: % time centre total: r_p_ = −0.89, p = 0.01).

**Table 1:** Correlations between running, open field behaviour and neurogenesis in exercising male C57Bl/6 J mice

**14d Runner**	**Distance**	**Rearing**	**%Time Centre**	**Running Distance**
	**Total**	**Total**	**Total**	
**Running Distance**		ns	ns	
***P *value**	**0.02**			-
***r***	**0.54**			
**Ki67**	ns	ns	ns	ns
***P *value**				
***r***				
**BrdU**	ns	ns	ns	ns
***P *value**				
***r***				
**# DCX**	ns	ns	ns	ns
***P *value**				
***r***				
**28d Runner**	**Distance**	**Rearing**	**%Time Centre**	**Running Distance**
	**Total**	**Total**	**Total**	
**Running Distance**		ns	ns	-
***P *value**	**0.01**			
***r***	**0.80**			
**# Ki67**	ns			ns
***P *value**		**0.09**	**0.01**	
***r***		**0.73**	**-0.89**	
**# BrdU**	ns	ns	ns	ns
***P *value**				
***r***				
**%BrdU/Calbindin**	ns	ns	ns	ns
***P *value**				
***r***				
**# DCX**	ns	ns	ns	ns
***P *value**				
***r***				

*r* = correlation coefficient.

## Discussion

Both 14- and 28-days of voluntary running in mice affected different aspects of open field behaviour, but neither exercise dose produced anxiolytic or anxiogenic effects. Moreover, the 28 day exercise dose did not produce changes in anxiety-related behaviour in the light/dark box test. Both durations influenced the various stages of neurogenesis with proliferation increased after 14 days, survival elevated after both 14 and 28 days and the rate of differentiation and maturation of newly born neurons increased in the 28-day runners compared to controls. Regression analysis identified a significant correlation between the proliferation marker and the anxiety-related parameter of total time spent in the centre of the open field but not with markers of survival, differentiation and maturation of adult born new neurons.

### Exercise and neurogenesis

Neural plasticity in the form of hippocampal neurogenesis is known to be potently increased subsequent to exercise in a cell population connected to hippocampal-related behaviours [26]. In terms of exercise-induced proliferation [1,16,27], enhanced survival of the young granule cells [3,16,28,29] and the increased rate of differentiation and maturation into neurons [3,30,31], our results are consistent with these foregoing studies.

### Exercise and behaviour

In the open field, the 28-day running mice moved significantly less than controls, suggesting decreased locomotor activity. A similar pattern was observed in the 14-day runners. Furthermore, both 14- and 28-day runners reared significantly more during the 20-minute test. One interpretation is that the running mice exhibited decreased reactivity to the aversiveness of the open field environment and increased exploration. A similar behavioural pattern has been observed in other tests in previous studies [32,33].

Concerning the classic open field index of anxiety; the amount of time spent in the centre of the arena, neither running period was anxiolytic or anxiogenic in either the 14- or 28-day runners. Although both running durations were previously shown to produce anti-anxiety effects in the open field [19,20], there are also other studies reporting increased anxiety or no change [17,31,34]. Furthermore, Fuss and co-workers (2010) demonstrated a similar decrease in locomotor activity in the open field without clear changes in anxiety-related behaviour; they report an effect of running on “distance to walls” during the second half of the 10 minute test (arguably an activity- rather than an anxiety-related measure) and do not describe other centre zone alterations. The open field was also the first of a list of tests they performed on their exercising mice; the increased anxiety was apparent in subsequent tests e.g. the light/dark box.

To assess whether no change in anxiety-related behaviour was due to a possible lack of sensitivity of the open field as a measure, we also performed the light/dark box test on a separate cohort of exercising mice. The outcome was consistent with the findings from the open field, i.e. no significant change in anxiety-related indices. Together, these results suggest that under these experimental conditions chronic voluntary wheel running is not anxiolytic or anxiogenic. Furthermore, there was no alteration between the control group without running wheel and the blocked wheel controls, indicating that the presence or absence of a (blocked) wheel did not matter for the anxiety-related behaviour of the control group.

### Correlation between exercise-induced hippocampal proliferation and anxiety

We found a significant negative correlation between anxiety in the open field (centre time) and the number of cells positive for Ki67, which labels proliferating cells that are still pluripotent and is thus not strictly a neuronal marker, in the dentate gyrus after 28 days running. This implies that the more proliferation of hippocampal precursor cells (that includes both neuronal and glial precursors), the more anxious the animal at this time-point (see Table 1). Importantly, we did not however observe a correlation between open field anxiety and either the survival (BrdU positive cell number) or maturation (% BrdU/Calbindin maturation rate) phases of neurogenesis. Thus, there was no apparent relationship between open field anxiety and mature newly formed neurons integrated into the existing hippocampal circuitry, which are capable of recruitment for behavioural functioning [35]. Fuss and co-workers (2010) observed a significant negative correlation between anxiety and neurogenesis after 6 weeks running; nevertheless, this was with doublecortin, a marker of newly born cells that have already differentiated into neurons. Furthermore, doublecortin levels were correlated to anxiety-measures assessed 19 to 23 days prior to the sacrifice of the animals, i.e. in contrast to our experiment, there was a time gap between the behavioural test and the assessment of neurogenic markers. Taken together, the fact that we did not see the correlation between anxiety and the rate of maturation of newly born neurons and no change in anxiety-related behaviour in the open field the day before sacrifice suggests that functionally mature neurons present at the time point of testing are not, in isolation, required for the genesis of anxiety per se as previously asserted by Fuss and co-workers (2010). An alternative explanation is that learning due to the repeated experience and/or prolonged time involved in the repeated behavioural testing in their study involved recruitment of the newly produced neurons and thereby underlies the correlation between maturation and anxiety-related behaviour.

Our result seems to conflict with a more recent study from the group of Fuss et al. [36]. They showed that irradiation blocked the anxiogenic effect of exercise, suggesting a role for neurogenesis in producing this behaviour. There are some possible explanations for the apparent conceptual discrepancy between the studies. For instance, a similar anxiety behavioural battery was used as in their previous study, testing open field first, followed by the O-maze and the light/dark box. Thus, the effect of repeated testing and the role played by neurogenesis under these circumstances cannot be excluded, as detailed above. Furthermore, while there appears to be an effect on anxiety in the first test, the open field (decreased centre time), this could be arguably secondary to the decreased locomotor activity in these mice. In addition, the use of irradiation to inhibit neurogenesis is not ideal due to possible side effects and this manipulation even appears to increase anxiety in the light/dark box. In sum, the findings of this particular study, while conflicting, are not conclusive

## Conclusions

Overall, our data show that while voluntary wheel running increases neurogenesis measures in mice, it does not produce an anxiolytic or anxiogenic effect in the open field. Most studies to date examining the relationship between neurogenesis and anxiety have been loss-of-function approaches [6,12,13]. The lack of an effect of exercise on anxiety in our mice, combined with the robust concomitant increase in neurogenesis implies that, as distinct from the findings of Fuss et al [17], a pure gain-of-neurogenesis in normal mice does not necessarily alter anxiety-related behaviour without further stimulation. Taken together, this suggests that neurogenesis may be involved in the learning of anxiety, but does not produce anxiety *per se*.

## Abbreviations

BDNF: Brain-derived neurotrophic factor; BrdU: 5’-bromo-2’-deoxyuridine.

## Competing interests

The authors declare that they do not have any competing interests.

## Authors’ contributions

LG carried out the behavioural and immunohistochemical studies, performed the statistical analyses and drafted the manuscript. DCL participated in the conception and design of the study and in drafting the manuscript. MHdA was involved in the critical revision of the manuscript for the final draft. WW participated in the conception of the study and in the critical revision of the manuscript. SH conceived the study, participated in its design, coordination and interpretation and analysis of the results and helped to draft the manuscript. All authors read and approved the final manuscript.
